# Association of anxiety and depression with physical and sensory functional difficulties in adults in five population-based surveys in low and middle-income countries

**DOI:** 10.1371/journal.pone.0231563

**Published:** 2020-06-26

**Authors:** Sarah Wallace, Islay Mactaggart, Lena Morgon Banks, Sarah Polack, Hannah Kuper

**Affiliations:** 1 Public Health Training Scheme, London Deanery, London, United Kingdom; 2 International Centre for Evidence in Disability, London School of Hygiene & Tropical Medicine, London, United Kingdom; Università degli Studi di Perugia, ITALY

## Abstract

**Background:**

The aim of this study was to assess the association between anxiety and depression with physical and sensory functional difficulties, among adults living in five low and middle-income countries (LMICs).

**Methods and findings:**

A secondary data analysis was undertaken using population-based disability survey data from five LMICs, including two national surveys (Guatemala, Maldives) and 3 regional/district surveys (Nepal, India, Cameroon). 19,337 participants were sampled in total (range 1,617–7,604 in individual studies). Anxiety, depression, and physical and sensory functional difficulties were assessed using the Washington Group Extended Question Set on Functioning. Age-sex adjusted logistic regression analyses were undertaken to assess the association of anxiety and depression with hearing, visual or mobility functional difficulties.

The findings demonstrated an increased adjusted odds of severe depression and severe anxiety among adults with mobility, hearing and visual functional difficulties in all settings (with ORs ranging from 2.0 to 14.2) except for in relation to hearing loss in India, the Maldives and Cameroon, where no clear association was found. For all settings and types of functional difficulties, there was a stronger association with severe anxiety and depression than with moderate. Both India and Cameroon had higher reported prevalences of physical and sensory functional difficulties compared with Nepal and Guatemala, and weaker associations with anxiety and depression.

**Conclusion:**

People with physical and sensory functional difficulties are more likely to report experiencing depression and anxiety. This evidence supports the need for ensuring a good awareness of mental health among those working with individuals with physical and sensory functional difficulties in LMICs. This implies that these practitioners must have the skills to identify anxiety and depression. Furthermore, mental health services must be available and accessible to patients with these conditions, which will likely require further programmatic scale-up in these LMIC settings.

## Introduction

Disability is an umbrella term, including people, covering functional difficulties (impairments), activity limitations, and participation restrictions, inclusive of mental health conditions [1]. The World Health Organization’s 2011 Global Report on Disability estimates that 15% of people experience significant functioning difficulties in their everyday lives [1]. However, people with disabilities may experience functional difficulties in multiple domains. Indeed, it is well-established that poor physical health can have a deleterious effect on other aspects of a person’s wellbeing [2–4], and that people with physical and/or sensory functional difficulties are at higher risk of mental health conditions, including anxiety and depression [5, 6]. For instance, studies across the globe have demonstrated that people with visual impairment are more likely to experience depression [7, 8], and there appears to be a dose-response relationship with more severe visual impairment linked to a higher prevalence of depression [9]. People with physical impairments or hearing loss are also more likely to report poorer mental health, including depression [3, 10–14]. The likely pathway for these associations is that people with sensory or physical impairments experience difficulties in activities and functioning, which have consequences for reduced perceived control, lower social connectedness, and worse financial circumstances, which all increase the risk of depression and other mental health conditions [2, 15–17]. The presence of pain may also contribute to the link between impairments with depression, for instance among people with functional difficulties [18, 19].

The higher prevalence of depression and other mental health conditions in people with other types of disabilities is an important issue. First, as already described, approximately 15% of people experience significant functioning difficulties in their everyday lives [1]. Furthermore, people with impairments who experience depression have lower quality of life than those without these mental health impacts [20]. The association of physical/sensory functional difficulties with depression and anxiety has implications for advocacy and service planning. Rather than viewing psychosocial and other impairments in isolation, it is vital to understand these interactions and support people holistically, although this will require scaling up of mental health services, particularly in LMICs [21]. It is also important to identify factors that are associated with an increased risk of poor mental health among people with other types of disabilities to inform areas for intervention.

However, there are important gaps in knowledge on the link between physical and sensory functional difficulties and poor mental health. There has been little work to compare how different functional limitations (e.g. hearing, vision, or physical) are associated with anxiety and depression, even though these relationships are likely to vary. Moreover, most studies in the existing evidence base have focussed on the link of depression with functional limitations, rather than anxiety [3]. Another gap is that data from LMICs is sparse [5, 13], and often does not distinguish between types of functional limitations [22, 23]. There are also concerns that existing studies may be prone to bias, and variations between studies in the ways in which mental and physical conditions are measured makes it difficult to compare data across settings [6].

In this study, we aimed to assess the association between anxiety and depression and physical and sensory (vision or hearing) functional difficulties, among adults living in LMICs, using data from five population-based surveys. This secondary data analysis was undertaken to support a better understanding of the comorbidity between mental health and other impairments, and therefore promote better integrated health services.

## Materials and methods

### Population-based surveys

This study uses data from five cross-sectional surveys undertaken between 2011 and 2017 in five LMICs: Cameroon, Guatemala, Maldives, India and Nepal (Table 1) [24–26]. Three were regional/district level surveys, while two (Guatemala, Maldives) were national surveys. All surveys used population-based sampling, and the response rates ranged between 82% and 95%. While the original surveys included children, our analyses are restricted to adults ≥18 years. Individuals (or representatives if unable to self-report) were asked a series of questions, of which the reported Washington Group Extended Question Set on Functioning (Box 1) formed part. The Washington Group Questions are a validated and widely used tool to screen for functional difficulties [27].

**Table 1:** Survey participants (≥18 years).

	Nepal	Guatemala	India	Cameroon	Maldives
Place, date	Tanahun, 2016	National, 2016	Mahbubnagar District, Telengana State, 2014	Fundong Health District (North West), 2013	National, 2017
Size (response rate %*)	4,067 (95%)	7,604 (88%)	2,349 (88%)	1,617 (87%)	3,700 (82%)
Average age (years), range	43.0 (18–96)	39.2 (18–100)	39.2 (18–98)	44.5 years (18–99)	39.3 years (18–102)
% Female	57.7%	57.0%	54.3% (1,277)	69.8% (1,129)	57.5% (2,147)
% Rural^α^	73.1%	58.2%	Not available	Not available	61.5%

*Response rate was for the whole survey whereas everything else is quoted for adults only.

^α^ Defined as outside of the capital Male’ for the Maldives.

**Box 1. Relevant Washington Group Extended Question Set on Functioning Questions (source: Washington group on disability statistics [9])**

VISION

1 Do you wear glasses? (Yes/No)
2 If yes, do you have difficulty seeing even when wearing your glasses? (No difficulty / Some difficulty /A lot of difficulty /Cannot do at all or unable to do).
3 If no, do you have difficulty seeing? (No difficulty / Some difficulty /A lot of difficulty /Cannot do at all or unable to do).

HEARING

4 Do you wear a hearing aid? (Yes/No)
5 If yes, do you have difficulty hearing even when using a hearing aid? (No difficulty / Some difficulty /A lot of difficulty /Cannot do at all or unable to do).
6 If no, do you have difficulty hearing? (No difficulty / Some difficulty /A lot of difficulty /Cannot do at all or unable to do).

MOBILITY

7 Do you use any equipment or receive help for getting around? (Yes/No)
8 If yes, Do you have difficulty walking or climbing steps, even when using your equipment or with help? (No difficulty / Some difficulty /A lot of difficulty /Cannot do at all or unable to do).
9 Do you have difficulty walking or climbing steps? (No difficulty / Some difficulty /A lot of difficulty /Cannot do at all or unable to do).

OR (For Guatemala only)

10 Do you have difficulty walking or climbing steps? (No difficulty / Some difficulty /A lot of difficulty /Cannot do at all or unable to do)
11 Do you use any equipment or receive help for getting around? (Yes/No)

ANXIETY

12 How often do you feel worried, nervous or anxious? (Daily/ Weekly/ Monthly/ A few times a year/ Never)
13 Do you take medication for these feelings? (Yes/No) (Not Nepal)
14 Thinking about the last time you felt worried, nervous or anxious, how would you describe the level of these feelings? (A little/ A lot/ Somewhere in between a little and a lot).

DEPRESSION

15 How often do you feel depressed? (Daily/ Weekly/ Monthly/ A few times a year/ Never)
16 Do you take medication for these feelings? (Yes/No) (Not Nepal)
17 Thinking about the last time you felt depressed, how would you describe the level of these feelings? (A little/ A lot/ Somewhere in between a little and a lot).

### Classification of functional difficulty

Box 1 details the Washington group questions that were used in this study. Individuals were classed as having a functional difficulty (vision, hearing or mobility) if they answered ‘a lot of difficulty’ or ‘cannot do at all’/‘unable to do’ to the relevant question, and no difficulty if they answered ‘none’ or ‘some’. This classification was based on their function ‘with aid’ (i.e glasses, hearing aids or mobility devices) if the individual reported use of aids, and ‘without aid’ if no use of aids was reported. There were some small variations in the questions used, for example in the Guatemala study the mobility question was not dependent on using an aid. Our hypothesis is that functional difficulty may lead to poor mental health. Consequently, the remaining questions from the Washington Group Short Set, on understanding, self-care and communication, were not included in our analyses as these are potentially consequences of poor mental health.

### Classification of anxiety and depression

**Fig 1:**
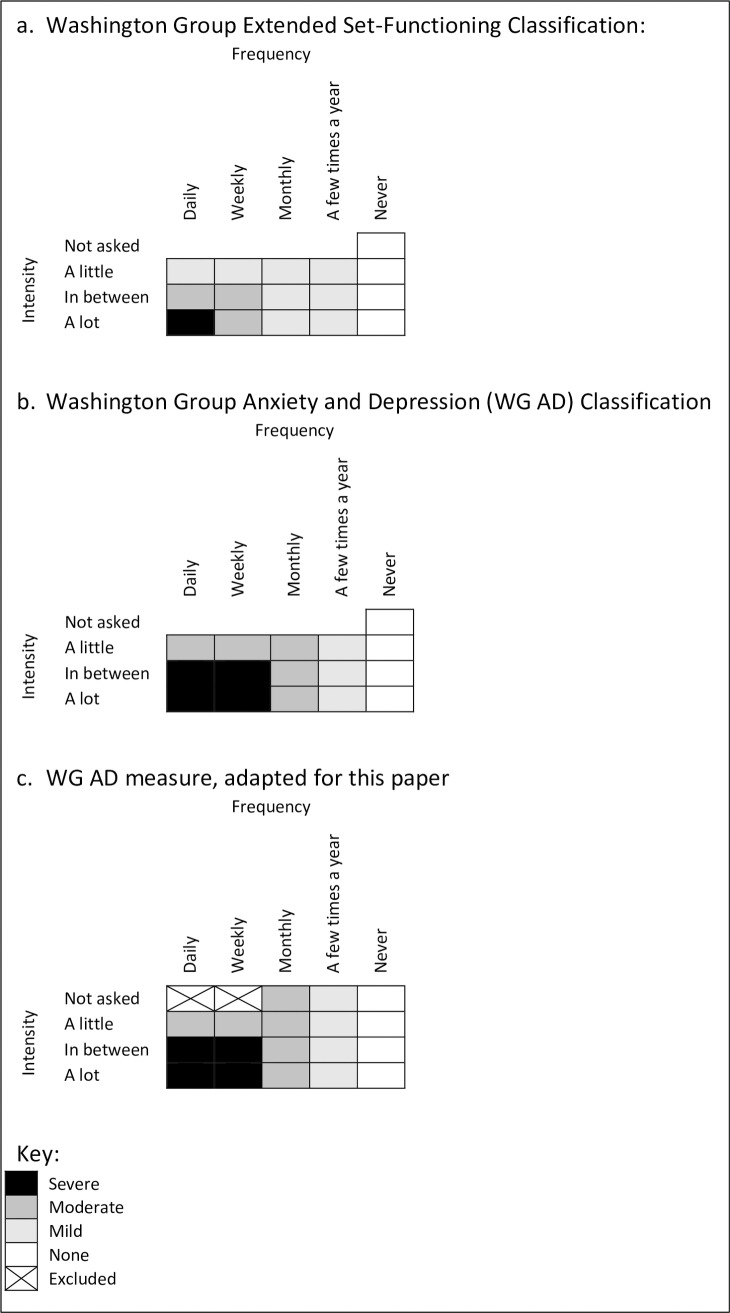
Anxiety and depression classification using Washington Group Questions (source: Washington Group Mental Health Work Group Update [28]).

There are two questions used to classify each of anxiety and depression (Box 1) which asked about frequency and severity of symptoms; only individuals who answered daily, weekly, monthly or a few times a year were then asked about severity. The more recent ‘Washington Group Anxiety and Depression’ (WG AD) definition was used to classify individuals as severe, moderate, mild/none for anxiety and depression rather than the original ‘Washington Group Extended Set-Functioning’ (WG ES-F) classification (Fig 1) [28]. When there was missing data, we used responses for the frequency question to determine the anxiety and depression level where there was only one classification possible (see Fig 1C. WG AD measure, adapted for this paper).

### Statistical analysis

Stata 15 was used for statistical analysis. Multivariable logistic regression was undertaken to assess the association between each of the three domains of functional difficulty (vision, hearing, mobility) with anxiety and depression, separately for each data set. The regression analyses were adjusted for age group (18–44, 45–65 and 65+ groups) and sex. A small number of participants had missing data for individual functioning questions, and these responses were retained and used within the data analysis where possible.

### Ethics and consent

Each survey received separate approval from the London School of Hygiene & Tropical Medicine and from local ethics committees [24–26]. Written (signed or fingerprint) informed consent was obtained from all subjects.

## Results

A total of 19,337 adults (age 18+ years) were included in the analysis; the largest survey was Guatemala (7,604) and the smallest was Cameroon (1,617). The prevalence of functional difficulty varied between the five surveys by domain: hearing (report ‘cannot do’ or ‘a lot’ of difficulty) ranged between 1.0% and 3.5%, visual 0.7% to 3.6% and mobility 2.0% to 5.5% (Table 2). Hearing aids were rare in all settings (0.1% to 0.6% of the whole surveyed population of adults), while glasses (6.3% to 35.7%) and mobility aids (2.2% to 16.1%) were much more common (S1 Table).

**Table 2:** Prevalence of different domains of functional difficulty.

		Nepal	Guatemala	India	Cameroon^$^	Maldives
Hearing*						
”A lot or more”	n	53	120	86	33	36
	% (95% CI)	1.3% (1.0–1.7)	1.6% (1.3–1.9)	3.7% (3.0–4.5)	2.0% (1.5–2.9)	1.0% (0.7–1.3)
Visual*						
”A lot or more”	n	27	240	85	48	86
	% (95% CI)	0.7% (0.5–1.0)	3.2% (2.8–3.6)	3.6% (2.9–4.5%)	3.0% (2.3–3.9)	2.3% (1.9–2.9%)
Mobility*						
”A lot or more”	n	83	214	111	88	166
	% (95% CI)	2.0% (1.6–2.5)	2.8% (2.5–3.2)	4.7% (3.9–5.7)	5.5% (4.5–6.7)	4.5% (3.9–5.2)
**Anxiety**						
Severe	n	47	466	181	94	304
	% (95% CI)	1.2% (0.9–1.6)	6.1% (5.6–6.7)	7.8% (6.7–8.9)	5.9% (4.8–7.1)	8.2% (7.4–9.1)
Moderate	n	175	1,610	457	410	208
	% (95% CI)	4.4% (3.8–5.1)	21.2% (20.2–22.1)	19.6% (18.0–21.3)	25.6% (23.5–27.8)	5.6% (4.9–6.4)
Mild/None	n	3,760	5,525	1,694	1,097	3,188
	% (95% CI)	94.4% (93.7–95.1)	72.7% (71.7–73.7)	72.6% (70.8–74.4)	68.5% (66.2–70.8)	86.2% (85.0–87.2)
**Depression**						
Severe	n	35	236	141	75	244
	% (95% CI)	0.9% (0.6–1.2)	3.1% (2.7–3.5)	6.1% (5.2–7.1)	4.8% (3.9–6.0)	6.6% (5.8–7.4)
Moderate	n	195	991	376	299	162
	% (95% CI)	4.9% (4.3–5.6)	13.0% (12.3–13.8)	16.2% (14.7–17.7)	19.3% (17.4–21.3)	4.4% (3.8–5.1)
Mild/None	n	3,746	6,371	1,811	1,177	3,294
	% (95% CI)	94.2% (93.4–94.9)	83.9% (83.0–84.7)	77.8% (76.1–79.4)	75.9% (73.7–78.0)	89.0% (88.0–90.0)

*Available correction or assistance (i.e. reported using hearing aids, glasses, or walking aids if the respondent has these aids).

^$^1 individual answered ‘don’t know’ for hearing, and 1 individual for mobility.

Severe anxiety ranged from 1.2% prevalence in Nepal to 8.2% in the Maldives, while moderate levels were between 4.4% (Nepal) and 25.6% (Cameroon) (Table 2). Depression prevalence showed the same pattern; it was lowest again in Nepal (severe 0.9%, moderate 4.9%) and highest in the Maldives for severe depression (6.6%) and in Cameroon for moderate (19.3%). Across the settings, few participants were taking medication for anxiety (from 0.9% of the whole surveyed population of adults in India, to 9.5% in Guatemala), or depression (0.6% to 3.1%) (S1 Table).

Tables 3, 4 and 5 show the prevalence of high, medium and low/no anxiety and depression by type of functional difficulty. Self-reported anxiety and depression prevalence were highest in Guatemala for all functional difficulty types, and lowest in Nepal (vision and mobility) and Cameroon (hearing). For all countries except Nepal and the Maldives, individuals reporting mobility difficulties were found to have the highest prevalence of severe anxiety and depression.

**Table 3:** Association between anxiety and depression and functional difficulties in hearing*.

	People with hearing functional difficulties (a lot or cannot do)	People without hearing functional difficulties (no difficulty/some difficulty)	OR (95% CI) adjusted for age and sex	P-value
**Nepal**	**N = 48**	**N = 3,934**	**N = 3,982**	
Anxiety				
• Severe	10.4%	1.1%	9.6 (3.5, 26.6)	<0.001
• Moderate	10.4%	4.3%	2.4 (0.9, 6.2)	0.08
• Mild/None	79.2%	94.6%	Baseline	
Depression				
• Severe	8.5%	0.8%	11.7 (3.8, 36.5)	<0.001
• Moderate	21.3%	4.7%	4.9 (2.3, 10.2)	<0.001
• Mild/None	70.2%	94.5%	Baseline	
**Guatemala**	**n = 120**	**n = 7,481**	**n = 7,600**	
Anxiety				
• Severe	25.0%	5.8%	4.9 (3.1, 7.8)	<0.001
• Moderate	22.5%	21.2%	1.5 (0.9, 2.3)	0.11
• Mild/None	52.5%	73.0%	Baseline	
Depression				
• Severe	20.0%	2.8%	6.3 (3.8, 10.4),	<0.001
• Moderate	14.2%	13.0%	1.3 (0.7, 2.2)	0.38
• Mild/None	65.8%	84.1%	Baseline	
**India**	n = 85	n = 2,247	n = 2,332	
Anxiety				
• Severe	15.3%	7.5%	1.7 (0.9, 3.2)	0.12
• Moderate	21.2%	19.5%	1.0 (0.6, 1.7)	0.90
• Mild/None	63.5%	73.0%	Baseline	
Depression				
• Severe	13.4%	5.8%	1.9 (0.9, 3.8)	0.07
• Moderate	18.3%	16.1%	1.0 (0.6, 1.8)	0.97
• Mild/None	68.3%	78.1%	Baseline	
**Cameroon**	n = 32	n = 1,567	n = 1,599	
Anxiety				
• Severe	9.4%	5.8%	1.4 (0.4, 5.0)	0.56
• Moderate	25.0%	25.7%	1.1 (0.5, 2.5)	0.84
• Mild/None	65.6%	68.5%	Baseline	
Depression				
• Severe	6.5%	4.8%	0.9 (0.2, 4.2)	0.94
• Moderate	16.1%	19.4%	0.8 (0.3–2.2)	0.71
• Mild/None	77.4%	75.8%	Baseline	
**Maldives**	n = 36	n = 3,664	n = 3,700	
Anxiety				
• Severe	22.2%	8.1%	2.8 (1.2–6.5)	0.02
• Moderate	2.8%	5.7%	0.5 (0.1–4.2)	0.56
• Mild/None	75.0%	86.3%	Baseline	
Depression				
• Severe	13.9%	6.5%	2.1 (0.8–5.7)	0.15
• Moderate	5.6%	4.4%	1.7 (0.4–7.5)	0.50
• Mild/None	80.6%	89.1%	Baseline	

* There were some missing data for depression variables: 3–5 people within Nepal, Guatemala, and India across cases and controls, and 1 case and 49 controls in Cameroon.

**Table 4:** Association between anxiety and depression and functional difficulties in vision*.

	People with visual functional difficulties (a lot or cannot do)	People without visual functional difficulties (no difficulty/ some difficulty)	OR (95% CI) adjusted for age and sex	p-value
**Nepal**	**n = 24**	**n = 3,958**	**n = 3,982**	
Anxiety				
• Severe	8.3%	1.1%	7.8 (1.7, 35.5)	0.01
• Moderate	20.8%	4.3%	5.4 (1.9, 15.1)	0.001
• Mild/None	70.1%	94.6%	Baseline	
Depression				
• Severe	8.7%	0.8%	13.8 (2.9, 65.6)	0.001
• Moderate	34.8%	4.7%	10.1 (4.0, 25.2)	<0.001
• Mild/None	56.5%	94.4%	Baseline	
**Guatemala**	**n = 240**	**n = 7,361**	**n = 7,600**	
Anxiety				
• Severe	30.4%	5.3%	8.8 (6.3, 12.2)	<0.001
• Moderate	27.1%	21.0%	2.2 (1.6, 3.0)	<0.001
• Mild/None	42.5%	73.7%	Baseline	
Depression				
• Severe	20.4%	2.5%	9.3 (6.4, 13.6)	<0.001
• Moderate	22.5%	12.7%	2.4 (1.7, 3.4)	<0.001
• Mild/None	57.1%	84.7%	Baseline	
**India**	**n = 84**	**n = 2,246**	**n = 2,330**	
Anxiety				
• Severe	16.7%	7.4%	2.0 (1.1, 3.9)	0.03
• Moderate	27.4%	19.3%	1.3 (0.8, 2.3)	0.28
• Mild/None	56.0%	73.3%	Baseline	
Depression				
• Severe	17.9%	5.6%	3.2 (1.7, 6.1)	<0.001
• Moderate	25.0%	15.8%	1.7 (1.0, 3.0)	0.05
• Mild/None	57.1%	78.6%	Baseline	
**Cameroon**	**n = 48**	**n = 1,544**	**n = 1,592**	
Anxiety				
• Severe	14.6%	5.6%	2.6 (1.1, 6.4)	0.04
• Moderate	25.0%	25.5%	1.2 (0.6, 2.4)	0.62
• Mild/None	60.4%	68.9%	Baseline	
Depression				
• Severe	20.0%	5.9%	3.4 (1.5, 7.5)	0.004
• Moderate	17.8%	17.7%	1.2 (0.5, 2.8)	0.599
• Mild/None	62.2%	76.4%	Baseline	
**Maldives**	**n = 86**	**n = 3,614**	**n = 3,700**	
Anxiety				
• Severe	30.2%	7.7%	5.7 (3.4–9.5)	<0.001
• Moderate	10.5%	5.5%	3.0 (1.4–6.3)	0.004
• Mild/None	59.3%	86.8%	Baseline	
Depression				
• Severe	30.2%	6.0%	7.8 (4.6–13.1)	<0.001
• Moderate	9.3%	4.3%	4.1 (1.9–9.1)	<0.001
• Mild/None	60.5%	89.7%	Baseline	

* There were some missing data for depression variables: 3–4 people within Nepal, Guatemala, and India across cases and controls, and 3 cases and 53 controls in Cameroon.

**Table 5:** Association between anxiety and depression and functional difficulties in mobility.

	People with mobility functional difficulties (a lot or cannot do)	People without mobility functional difficulties (no difficulty/ some difficulty)	OR (95% CI) adjusted for age and sex	p-value
**Nepal**	**n = 79**	**n = 3,903**	**n = 3,982**	
Anxiety				
• Severe	10.1%	1.0%	12.6 (5.4, 29.5)	<0.001
• Moderate	21.5%	4.1%	7.2 (4.0, 13.1)	<0.001
• Mild/None	68.4%	95.0%	Baseline	
Depression				
• Severe	7.6%	0.7%	14.2 (5.4, 37.1)	<0.001
• Moderate	29.1%	4.4%	9.6 (5.5, 16.4)	<0.001
• Mild/None	63.3%	94.8%	Baseline	
**Guatemala**	**n = 214**	**n = 7,387**	**n = 7,600**	
Anxiety				
• Severe	34.1%	5.3%	10.4 (7.3, 14.7)	<0.001
• Moderate	24.3%	21.1%	2.0 (1.4, 2.9)	<0.001
• Mild/None	41.6%	73.6%	Baseline	
Depression				
• Severe	25.7%	2.5%	13.9 (9.4, 20.6)	<0.001
• Moderate	22.4%	12.8%	2.7 (1.9, 3.8)	<0.001
• Mild/None	51.9%	84.8%	Baseline	
**India**	**n = 111**	**n = 2,221**	**n = 2,332**	
Anxiety				
• Severe	22.5%	7.0%	4.0 (2.3, 6.9)	<0.001
• Moderate	28.8%	19.1%	1.8 (1.1, 2.9)	0.02
• Mild/None	48.7%	73.8%	Baseline	
Depression				
• Severe	22.5%	5.2%	5.4 (3.1, 9.4)	<0.001
• Moderate	23.4%	15.8%	1.7 (1.0, 2.8)	0.04
• Mild/None	54.1%	79.0%	Baseline	
**Cameroon**	**n = 87**	**n = 1,505**	**n = 1,592**	
Anxiety				
• Severe	17.2%	5.2%	4.4 (2.3, 8.7)	<0.001
• Moderate	32.2%	25.2%	1.9 (1.2, 3.2)	0.01
• Mild/None	50.6%	69.6%	Baseline	
Depression				
• Severe	19.0%	5.5%	4.5 (2.3, 8.8)	<0.001
• Moderate	27.9%	17.4%	2.5 (1.4, 4.3)	0.001
• Mild/None	53.2%	77.2%	Baseline	
**Maldives**	**n = 166**	**n = 3,534**	**n = 3,700**	
Anxiety				
• Severe	25.9%	7.4%	5.2 (3.4–7.9)	<0.001
• Moderate	7.2%	5.6%	2.0 (1.0–3.8)	0.05
• Mild/None	66.9%	87.1%	Baseline	
Depression				
• Severe	25.3%	5.7%	7.7 (4.9–12.1)	<0.001
• Moderate	6.6%	4.3%	3.2 (1.6–6.6)	0.001
• Mild/None	68.1%	90.0%	Baseline	

* There were some missing data for depression variables: 3–6 people within Nepal, Guatemala, and India across cases and controls, and 8 cases and 41 controls in Cameroon.

In Nepal and Guatemala, there was strong evidence of an association between functional difficulties in hearing and severe anxiety and depression (Table 3). In India, the Maldives and Cameroon there was no evidence of an association between hearing difficulties and severe anxiety or depression. Moderate depression was only associated with hearing loss in Nepal, and moderate measures of depression or anxiety were unrelated to functional difficulties with hearing in the other settings.

Functional difficulties in vision were associated with severe anxiety and depression in all five settings (Table 4). Additionally, functional vision difficulties were associated with moderate anxiety and depression in Nepal, the Maldives and Guatemala, and moderate depression in India, but not with anxiety in India or either mental health condition in Cameroon.

Mobility functional difficulties were associated with both moderate and severe depression and anxiety in all settings (Table 5).

Several consistent patterns were observed across the types of functional difficulty (Tables 3–5). In the multiple regression analysis, as the absolute numbers of individuals with functional difficulties were small, the adjusted odds ratios (OR) often had wide confidence intervals. In most settings and types of functional difficulties, the point estimate of the association of the three functional difficulties with anxiety and depression was greater than 1, with depression generally slightly greater than anxiety (difference not significant at the p = 0.05 level). The magnitude of association of the functional difficulty with ‘high’ levels of anxiety or depression compared to with low levels/none, was always greater than the association with ‘medium’ levels compared to low/none. More evidence of associations for anxiety and depression with all functional impairment types were seen in Nepal and Guatemala, compared to Cameroon and India, although the latter were smaller studies. The estimates for the Maldives generally fell in the middle range.

## Discussion

This study provides evidence from five low and middle-income settings that the presence of a physical or sensory functional difficulties is associated with both anxiety and depression. This association was observed in all five studies, except for with hearing loss in India, the Maldives and Cameroon, where no clear association was found. For all settings, there was a stronger association of all types of functional difficulties with high levels of anxiety and depression than with moderate levels.

Our findings of increased odds of depression and anxiety in those with functional difficulties was generally consistent with the previous literature, mainly from high income settings [2–4]. Few studies were identified from low and middle-income countries, but these too reported an association between depression and/or anxiety with functional difficulties [22, 23, 29]. We noted a stronger association of severe mental health conditions with other functional difficulties, compared to more moderate conditions. Similarly, the England psychiatric morbidity survey showed a trend of stronger association of functional difficulties in mobility, vision and hearing and anxiety/depression as the severity of the anxiety/depression increased [17]. Our analysis did not examine the association of mental health conditions with multiple functional difficulties or the severity of those difficulties. However, a large study among people aged over 65 in China identified that, among those with motor disabilities, having other co-morbid disabilities and having more severe motor disability were both associated with a higher risk of psychiatric co-morbidity (cognitive, affective, or behavioral disorders) [5].

The explanation of the differences in magnitude of the association between different settings seen is not clear, particularly the reason why weaker associations are seen where the overall prevalence of physical and sensory functional difficulties is higher. There may be a true difference in risk of poor mental health between the settings, for example associated with the opportunities available for disabled people or community attitude to disability. However, it may also be connected to cultural variation, in particular, a difference in likelihood of reporting functional difficulties or symptoms of anxiety and depression in the different settings. Another potential explanation is the option of ‘some’ within the Washington Group questions, and whether differences in translation could impact reported severities. For example, it is possible that people with a broader range of severity in their vision, hearing and mobility functional limitations were included in the “a lot/cannot do” group in Cameroon and India on account of the translation or other cultural factors, who may have been included in the “none/some” group in the other settings.

There are strengths and limitations to the study, in addition to those already outlined, which should be taken into account when interpreting the results. The main strengths to this study are i) that it included large, population-based data sets from five different countries, ii) the relatively uniform methodology enabled us to compare different countries and iii) the comprehensive questions allowed us to assess several functional domains at the same time. The survey methodology has been validated and all had high response rates (≥82%). Our main hypothesis was that physical and sensory difficulties can lead to anxiety and depression, potentially as a result of pain, reduced perceived control, activity restriction, impact on financial circumstances and changing social relationships [2, 17]. However, it is also possible that the presence of anxiety and depression may change individuals’ perception and reporting of their physical and sensory functioning, and therefore may lead to a bias in the responses, as postulated by researchers in the past [18]. The potential for bias is a particular concern since the Washington group questions were used to measure both mental health and functional difficulties through self-report and do not include objective clinical criteria. However, our previous analyses have shown a relatively strong association of clinical and subjective criteria for the higher thresholds of the Washington Group questions [26, 28]. An additional weakness of this study is that it is not able to answer these questions of causation directly as it was cross-sectional rather than longitudinal, and limited adjustment for factors such as socio-economic status means that the associations may be influenced by (residual) confounding. Information was not collected on age at onset of the disability, which may be an important determinant of the impact on mental health. For instance, we could hypothesise that if onset occurs at a younger age then people would have had more time to adapt and therefore will present with fewer mental health consequences [30]. Furthermore, we did not consider the impact of the cause of disability–potentially traumatic injuries, as an example, may have more mental health impacts than those related to illness. The study also lacked qualitative data, which would have allowed the nature of these associations to be explored further.

Despite these limitations, it does appear that the prevalence of mental health conditions is linked to the presence of physical/sensory functional difficulties. Access to rehabilitation and mental health services is frequently poor in LMICs [19], and there are well documented barriers to healthcare access for individuals with a disability [31, 32]. The association of mental health and physical/sensory functional difficulties implies that there should be more integrated care between rehabilitation/specialist care and mental health services, and that general/primary healthcare services should be aware of comorbidities and multiple support requirements. Furthermore, there should potentially be routine assessment of depression/anxiety in people with physical and sensory functional difficulties. This initiative may involve training community health workers or rehabilitation staff to identify anxiety and depression among patients with physical and sensory impairments. Additionally, Mental Health and Psychosocial Services must be accessible to people with physical and sensory functional difficulties, which may involve adjustment in how, where or by whom the services are offered. These findings also have implications for the design of future studies. Morris’ literature review commented that there is ‘a tendency in research practice to deliberately exclude people with physical impairments if the focus of research is mental health difficulties’, which would have implications on the generalisability of mental health research among those with physical and sensory impairments [2, 17, 18, 26, 28, 33]

## Conclusion

This evidence supports the need for ensuring a good awareness of mental ill health among those working with individuals with physical and sensory functional difficulties in LMICs. This implies that these practitioners must have the skills to identify anxiety and depression. Furthermore, mental health services must be available and accessible to patients with these conditions, which will likely require further programmatic scale-up in these LMIC settings.

## Supporting information

S1 Table Individuals reporting aids or medication use for anxiety or depression. (DOCX)
