# Supplementary material for: Association of anxiety and depression with physical and sensory functional difficulties in adults in five population-based surveys in low and middle-income countries
Source: PLoS One. 2020 Jun 26;15(6):e0231563. doi: 10.1371/journal.pone.0231563 (PMC7319598; doi:10.1371/journal.pone.0231563)
Supplement: S1 Table — (DOCX) [file pone.0231563.s001.docx]

Supplementary Table 1: Individuals reporting aids or medication use for anxiety or depression

|  | **Nepal** | **Guatemala** | **India** | **Cameroon** | **Maldives** |
| --- | --- | --- | --- | --- | --- |
| Hearing aids, n (% of those who answered question) | 11 (0.3%) | 27 (0.4%) | 3 (0.1%) | 2 (0.1%) | 23 (0.6%) |
| Glasses, n (% of those who answered question) | 480 (11.8%) | 930 (12.2%) | 234 (10.0%) | 101 (6.3%) | 1,320 (35.7%) |
| Mobility aids, n (% of those who answered question) | 91 (2.2%) | 188 (2.5%) | 102 (4.3%) | 274 (16.1%) | 83 (2.2%) |
| Medication for anxiety, n (% of whole sample) | Not asked | 719 (9.5%) | 20 (0.9%) | 72 (4.5%) | 94 (2.5%) |
| Medication for depression, n (% of whole sample) | Not asked | 214 (2.8%) | 15 (0.6%) | 50 (3.1%) | 60 (1.6%) |
